# The interrater reliability of a routine outcome measure for infants and pre-schoolers aged under 48 months: Health of the Nation Outcome Scales for Infants

**DOI:** 10.1192/bjo.2021.39

**Published:** 2021-04-22

**Authors:** Peter Brann, Gordana Culjak, Nick Kowalenko, Rosemary Dickson, Tim Coombs, Anne Sved Williams, Elisabeth Hoehn, Simon Davies, Margaret Hoyland, Philip Burgess

**Affiliations:** Eastern Health Child and Youth Mental Health Service, Australia; and Monash University, Australia; Australian Mental Health Outcomes and Classification Network, Health Education and Training Institute, Australia; and Sydney Medical School, University of Sydney, Australia; Department of Psychological Medicine, Sydney Children's Hospital Randwick, Sydney Children's Hospital Network, Australia; Child and Adolescent Mental Health Information Development Expert Advisory Panel, Australian Mental Health Outcomes and Classification Network, Health Education and Training Institute, Australia; Australian Mental Health Outcomes and Classification Network, Australia; and Illawarra Institute for Mental Health, Australia; University of Adelaide, Australia; and Women's and Children's Health Network, Australia; Queensland Centre for Perinatal and Infant Mental Health, Child and Youth Mental Health Service, Children's Health Queensland Hospital and Health Service, Australia; Child and Adolescent Mental Health Service, Western Australia Child and Adolescent Health Service, Australia; Child and Youth Mental Health Service, Children's Health Queensland Hospital and Health Service, Australia; Australian Mental Health Outcomes and Classification Network Analysis and Reporting, School of Public Health, Faculty of Medicine, The University of Queensland, Australia

**Keywords:** HoNOSI, mental health, outcome measure, infants, interrater reliability

## Abstract

**Background:**

A review of Australian mental health services identified a gap in routine outcome measures addressing social, emotional and behavioural domains for pre-schoolers and infants. The Child and Adolescent Mental Health Information Development Expert Advisory Panel Working Group developed the Health of the Nation Outcome Scales for Infants (HoNOSI), a clinician-reported routine outcome measure for use with those aged under 4 years. Prior psychometric testing showed that the HoNOSI was considered to show face validity, and that it met the standards for concurrent validity and internal consistency.

**Aims:**

We aimed to investigate the interrater reliability of the HoNOSI.

**Method:**

Forty-five infant mental health clinicians completed HoNOSI ratings on a set of five case vignettes.

**Results:**

Quadratic weighted kappa interrater reliability estimates showed the HoNOSI to have *Almost Perfect* interrater reliability for the HoNOSI total score. Of the 15 scales, one had *Moderate*, seven had *Substantial* and seven had *Almost Perfect* interrater reliability. Ten of the fifteen scales and the total score exceeded the COnsensus-based Standards for the Selection of Health Measurement INstruments criteria for interrater reliability (*κ_w_* ≥ 0.7).

**Conclusions:**

There has been a clear need for a routine outcome measure for use with infants and pre-schoolers. This study provides evidence of interrater reliability. The current findings, combined with the face and concurrent validity studies, support further examination of HoNOSI in real-world settings.

In 1990, Jenkins identified an urgent need for a system of indicators to enable clinicians to monitor and evaluate mental healthcare.^1^ One reason identified for not routinely using standard outcome measures was the lack of appropriate instruments.^2^ In 1998, Wing et al^3^ developed the Health of the Nation Outcome Scales (HoNOS), an instrument covering symptoms, functioning, relationships and environmental issues,^4,5^ which could be used routinely in the UK National Health Service to measure progress toward the target set by the UK Department of Health ‘to improve significantly the health and social functioning of mentally ill people’.^6^ Since then, the HoNOS and its adaptations for children and adolescents (HoNOSCA) and for those aged >65 years (HoNOS65+), have been officially adopted in England, Australia, New Zealand and other European countries.^7–10^

Gowers et al^11^ developed the HoNOSCA as a set of scales to be used in Child and Adolescent Mental Health Services.^12^ The HoNOSCA has been widely used.^4,13–19^ It was designed to be brief, have a similar structure to the HoNOS and provide a broad, quantitative measure of severity to measure a range of behavioural, symptomatic, social and impairment domains in children and adolescents and it has been found to have sound psychometric properties.^11,20,21^

In Australia, the National Outcomes and Casemix Collection (NOCC) was introduced ‘to provide a suite of measures that support clinical practice and comparisons across services and different consumer populations’.^19^ This saw the implementation of a range of outcome measures to routine clinical practice across age groups. At the core of the NOCC was the family of clinician-rated HoNOS. A review of NOCC^22^ identified a measurement gap for infants and pre-schoolers.

The Australian Child and Adolescent Mental Health Information Development Expert Advisory Panel (CAMHIDEAP)^23^ provides advice to the Australian government on mental health information development in Australian child and adolescent mental health services. Several members of CAMHIDEAP participated in a collaborative international review of the interrater reliability of HoNOSCA.^13^ This study also involved participants from the UK, Denmark, Norway and New Zealand. CAMHIDEAP members, including Dr Peter Brann, Dr Tim Coombs and Dr Sally Merry as the New Zealand representative, commenced conversations with the other participants in the HoNOSCA interrater reliability study about the development of a measure for infants and pre-schoolers, i.e. the Health of the Nation Outcome Scales for Infants (HoNOSI).^24^ Australia and New Zealand, through the CAMHIDEAP, commenced the measure development work, with the initial mapping of domains undertaken by Dr Sally Merry. The CAMHIDEAP had formed a working group to look broadly at routine outcome measures in the very young and supported by the results of the review of the NOCC, took ownership of the HoNOSI initiative, developing and refining a measure suitable for sector consultation and field trialling.

Face validity testing^25^ showed that the HoNOSI was perceived to fill a much needed gap in infant mental health outcome measurement for the 0–47 month age group (infants and pre-schoolers). Following subsequent concurrent validity testing,^26^ the CAMHIDEAP Working Group identified the need to test the interrater reliability of the HoNOSI.

## Aims

This HoNOSI field trial was designed to examine interrater reliability, exploring the degree of agreement among different raters when rating the same case vignettes with the HoNOSI.

## Method

A pilot study with three vignettes was conducted by a panel of CAMHIDEAP Working Group members, along with individuals with expertise in infant mental health or mental health measurement. The pilot study identified that the vignettes were substantially more severe than the real cases in the concurrent validity study. The vignettes’ severity was adjusted to accord with the real cases and two more vignettes were added. The current study used the resulting five vignettes. The age and gender of the vignettes were 4 months (female), 9 months (male), 3 years (female), 3 years (female) and 4 years (male). The vignettes can be found within the full HoNOSI Field Trial Report.^25^ Presenting problems included perceived agitation, aggressiveness (9 months), a paediatric in-patient referral with feeding problems, sensory underresponsiveness and maternal depression (4 months).

For the current study, participants with expertise in infant and pre-school mental health were asked to rate these vignettes. Ethics and research governance approval was obtained from the relevant ethics committee for participants from the Centre for Perinatal and Infant Mental Health and Child and Youth Mental Health Service in Queensland (HREC Ref: HREC/16/QRCH/424) , and for participants from the Eastern Health Child Youth Mental Health Service in Victoria (HREC Ref: LR19/040). A total of 45 people participated in the study; 26 from Queensland and 19 from Victoria. All participants received a brief online training session on the measure,^27^ covering areas such as the rating rules and inclusion and exclusion criteria. Upon providing consent via the online consent form, the five vignettes were independently rated online. Those making the ratings were presented with the vignette and then the HoNOSI glossary, from which the rater selected their preferred rating. Results were analysed with Stata statistical software, version 16.1 for Windows.^28^ Interrater reliability was assessed with quadratic weighted kappa. A copy of the vignettes is available from the corresponding author, upon request.

The HoNOSI^24^ is a 15-item set of scales, rated from 0 to 9, with 0 indicating no problem, 1 indicating a minor problem requiring no formal action, 2 indicating a mild problem, 3 indicating a problem of moderate severity, 4 indicating a severe to very severe problem and 9 indicating not known or not applicable.

The total score is calculated by summing the first 13 scales, which relate to infant mental health status. Missing data is treated as zero in calculating totals. As with the HoNOS family of measures, clinicians typically classify ratings as ‘clinically significant’^29^ if a problem area is rated as either a *Mild*, *Moderate* or *Severe to Very Severe* problem (i.e. a rating of 2, 3 or 4). A list of HoNOSI scales can be found in Appendix 1.

The strength of agreement between raters was measured against the COnsensus-based Standards for the Selection of Health Measurement INstruments (COSMIN).^30^ The COSMIN initiative^30^ was developed to provide guidance on the selection of outcome measures for clinical and research applications.^31^ The COSMIN includes a methodology for assessing the interrater reliability of measures,^30,32^ a taxonomy and definitions of measurement properties,^33^ checklists for assessing the methodological quality^34^ and criteria for good measurement properties.^31^ For measures constructed with ordinal ratings (i.e. the rating categories indicate the relative ordering of ‘clinical severity’), the gold standard for reporting is the weighted kappa,^32^ where reliability is *sufficient* if *κ_w_* ≥ 0.7. The standards outlined by Landis and Koch^35^ are commonly used in discussions of reliability and will also be reported to both facilitate comparison and support interpretation.

## Results

Of the 45 raters, 39 completed all five vignettes with no missing values. Five raters had a missing rating for one scale with one vignette and one rater had a missing rating for one scale with two vignettes. No HoNOSI scale ratings were rated as 9 (not known/not applicable). In accordance with the rating rules used by the HoNOSCA, all vignettes were able to have total scores calculated.^20^ Table 1 shows descriptive statistics and the HoNOSI total score distribution for each of the five case vignettes. The percentiles indicate the total score that occurred at that percentage of the distribution. For example, for Levi, a score of 17 was >10% of the total scores. A score of 18 was >25% of total scores. The 50th percentile is the median score. Percentiles aid in describing the distribution and spread of total scores for each vignette.

**Table 1:** Distribution of Health of the Nation Outcome Scales for Infants total score for the five vignettes

Vignette	Raters, *n*	Mean	s.d.	Minimum	Maximum	Percentiles				
						10th	25th	50th	75th	90th
1 – Levi (9 months)	45	20.7	3.9	14	31	17	18	20	22	27
2 – Lily (4 months)	45	17.8	4.4	12	31	13	15	17	20	23
3 – Chloe (3 years)	45	10.3	3.6	2	22	6	8	10	12	13
4 – Sandy (4 years)	45	31.1	4.0	26	42	26	28	31	33	35
5 – Sally (3 years)	45	37.6	4.3	27	46	31	36	37	40	43

All of the possible HoNOSI ratings (0, 1, 2, 3, 4) were utilised for each of the 15 scales in at least one vignette. This demonstrates that the full range of possible scores were used in these vignettes and would contribute to the interrater reliability estimate.

Interrater reliability was assessed by quadratic weighted kappa (*κ_w_*) estimates (Table 2). The COSMIN standard for acceptable interrater reliability (*κ_w_* ≥ 0.7)^31^ was exceeded by 10 of the 15 scales and by the total score. From Table 2, it can be seen that scales 7 and 15 were within 0.01 of the standard. Scales 11 and 14 were within 0.08 of the standard. The confidence intervals for those four scales are wide and overlap the standard. Scale 2 had a very wide confidence interval and was clearly lower than the standard.

**Table 2:** Interrater reliability quadratic weighted kappa estimates for vignettes

HoNOSI scale		Raters, *n*	*κ_w_*	*κ_w_* 95% CI lower	*κ_w_* 95% CI upper	Strength of agreement
1	Problems with disruptive behaviour/irritability/undercontrolled emotional regulation	45	0.86	0.77	0.94	Almost perfect
2	Problems with activity levels, joint and/or sustained attention	45	0.41	0.00	0.81	Moderate
3	Non-accidental self-injury or lack of self-protective behaviours	44	0.85	0.79	0.92	Almost perfect
4	Problems with feeding and eating behaviour	45	0.85	0.61	1.00	Almost perfect
5	Problems with developmental delays	45	0.85	0.76	0.95	Almost perfect
6	Problems with physical illness or disability	45	0.80	0.57	1.00	Substantial
7	Problems associated with regulation and integration of sensory processing	43	0.69	0.35	1.00	Substantial
8	Problems associated with sleep	45	0.85	0.57	1.00	Almost perfect
9	Problems with emotional and related symptoms or overcontrolled emotional regulation	44	0.80	0.55	1.00	Substantial
10	Problems with social reciprocity	45	0.87	0.76	0.98	Almost perfect
11	Problems with age-appropriate self-care and environmental exploration	45	0.62	0.27	0.98	Substantial
12	Problems with family life and relationships	44	0.81	0.64	0.98	Almost perfect
13	Problems with attending care, education and socialisation settings	44	0.71	0.27	1.00	Substantial
14	Problems with knowledge or understanding about the nature of the infant's difficulties	45	0.66	0.34	0.97	Substantial
15	Problems with lack of information, understanding about services or managing the infant's difficulties	44	0.69	0.49	0.88	Substantial
HoNOSI total score		45	0.85	0.70	1.00	Almost perfect

HoNOSI, Health of the Nation Outcome Scales for Infants.

The Landis and Koch Strength of Agreement criteria^35^ is shown in the rightmost column. Scale 2 (activity/attention) is only *Moderate* in agreement (0.41–0.60). However, seven of the scales and the total score have *Almost Perfect* agreement (0.81–1.00), whereas the remaining seven have *Substantial* agreement (0.61–0.80).

## Discussion

This study was designed to test the interrater reliability of the HoNOSI. Results show that the level of interrater reliability for the total score was *Almost Perfect* when measured against the benchmarks outlined by Landis and Koch^35^ and that it also well exceeds the COSMIN standard for interrater reliability.^32^ The majority of individual scales clearly met the COSMIN criteria and applying the commonly used Landis and Koch^35^ descriptors, seven scales and the total score had *Almost Perfect* agreement and seven scales had *Substantial* agreement between raters.

COSMIN guidelines describe what constitutes a *sufficient* level of agreement. Using the COSMIN criteria for good measurement properties, the results of the present study well exceed the criteria for interrater reliability, where reliability is rated as either sufficient (*κ_w_* ≥ 0.7), insufficient (*κ_w_* < 0.7) or indeterminate (where *κ_w_* is not reported. Scales 7 (problems associated with regulation and integration of sensory processing), 11 (problems with age-appropriate self-care and environmental exploration), 14 (problems with knowledge or understanding about the nature of the infant's difficulties) and 15 (problems with lack of information, understanding about services or managing the infant's difficulties) were classed as insufficient in terms of reliability (Table 2), although they are close to the arbitrary cut-off (*κ_w_* ≥ 0.7).

It is only scale 2 (Problems with activity levels, joint and/or sustained attention), with *κ_w_* < 0.5, that was found to be insufficient according to the COSMIN criteria, although assessed as a *moderate* level of agreement according to the guidelines provided by Landis and Koch.^35^ Aside from scale 2, the other 14 HoNOSI scales and the total score showed a reasonable amount of variability between vignettes. The study had sufficient power to test the null hypothesis at the interrater reliability estimate of 0.5.

The HoNOSI has links with the HoNOS family of measures, especially the HoNOSCA.^11^ The HoNOSI estimates are stronger than those obtained with those measures.^13,15^ Although the HoNOSCA covers different developmental stages in its use across ages 4–18 years, the HoNOSI, with a much a narrower age range, covers developmental shifts of arguably greater magnitude. Although the interrater reliability was *Substantial* to *Almost Perfect* for the majority of the scales and the total score, it would be wise to establish whether this overall acceptable estimate hides less acceptable interrater reliability for different age groups. The lower estimate for attention may reflect a differential functioning of the scale for different age groups. It is possible that the scale can be applied very reliably for 9-month-olds and 3-year-olds, but less so for 18-month-olds.

Vignettes have been found to be a valid tool when measuring the quality of clinical practice.^36^ They remain a key method when wanting to ensure that raters are exposed to exactly the same stimuli. However, vignettes are not the same as clinical practice, and the HoNOSI may perform better or worse with real cases. Although the vignettes covered ages from 4 months to 4 years, there were only five presentations assessed. The middle age group was not covered. Evaluating the HoNOSI in routine clinical practice would supplement the vignette approach by ensuring that a greater range of presentations could be examined. Furthermore, the question of whether the HoNOSI has differing reliability between, for example, infants and pre-schoolers, could be examined with larger sampling.

It is anticipated that future research could explore HoNOSI psychometric properties with respect to other domains and consumer attributes, including the specific nature of presenting problems, diagnostic categories and developmental differences. With regard to the rapid maturation that occurs in this age range, conceptualising and assessing test–retest reliability will be an important task. A face validity study^25^ and concurrent validity field trial^26^ have now been completed. There are other psychometric properties (e.g. sensitivity to change) yet to be investigated. However, the interrater reliability findings are sufficiently encouraging to support the further exploration of the HoNOSI. The HoNOSI is promising, and the very young have been excluded from the routine outcome measurement framework for too long. Whether it proves to be useful in this endeavour requires further exploration of reliability, validity and feasibility and this may be best achieved by examination in routine clinical practice.

A final caveat: if the HoNOSI continues to demonstrate good enough psychometric properties in future research, it will still share one common feature with other routine outcome measures. Measured change cannot specify the cause of the change. The HoNOSI, HoNOSCA and similar measures identify change but do not independently specify whether this is clearly maturational, treatment-initiated or a result of alteration in the child's environment. Understanding the source of any change, positive or negative, remains the clinical task at an individual level and a research and policy task at the aggregate level. Hopefully, the HoNOSI will facilitate routine dialogue about mental health outcomes for our relatively overlooked infants and pre-schoolers.

## Data Availability

The data that support the findings of this study are available from the corresponding author, G.C., upon reasonable request.

## Appendix 1: Health of the Nation Outcome Scales for Infants (HoNOSI) – list of scales

**Table:**

Scale 1	Problems with disruptive behaviour/irritability/undercontrolled emotional regulation
Scale 2	Problems with activity levels, joint and/or sustained attention
Scale 3	Non-accidental self-injury or lack of self-protective behaviours
Scale 4	Problems with feeding and eating behaviour
Scale 5	Problems with developmental delays
Scale 6	Problems with physical illness or disability
Scale 7	Problems associated with regulation and integration of sensory processing
Scale 8	Problems associated with sleep
Scale 9	Problems with emotional and related symptoms or overcontrolled emotional regulation
Scale 10	Problems with social reciprocity
Scale 11	Problems with age-appropriate self-care and environmental exploration
Scale 12	Problems with family life and relationships
Scale 13	Problems with attending care, education and socialisation settings
Scale 14	Problems with knowledge or understanding about the nature of the infant's difficulties
Scale 15	Problems with lack of information, understanding about services or managing the infant's difficulties
